# Foetal inguinoscrotal hernia—its prenatal diagnosis and its spontaneous regression

**DOI:** 10.1259/bjrcr.20150277

**Published:** 2016-02-18

**Authors:** Anwer Sadat Kithir Mohamed

**Affiliations:** Radiology Department, Tiruvarur Medical Centre, Tiruvarur, India

## Abstract

A foetal inguinoscrotal hernia is a rare abnormality. We report a case of foetal inguinoscrotal hernia diagnosed by ultrasonography at 37 weeks gestation and its spontaneous regression during the perinatal period. The imaging features, differential diagnosis, pregnancy management and outcome are discussed.

## Case presentation

A 22-year-old female in her first pregnancy was referred to our centre at 37 weeks menstrual age for obstetric ultrasonography to assess foetal wellbeing. The clinical history was unremarkable. Routine obstetric sonographic examination at 22 weeks gestation did not reveal any abnormality.

## Investigation

**Figure 1.**
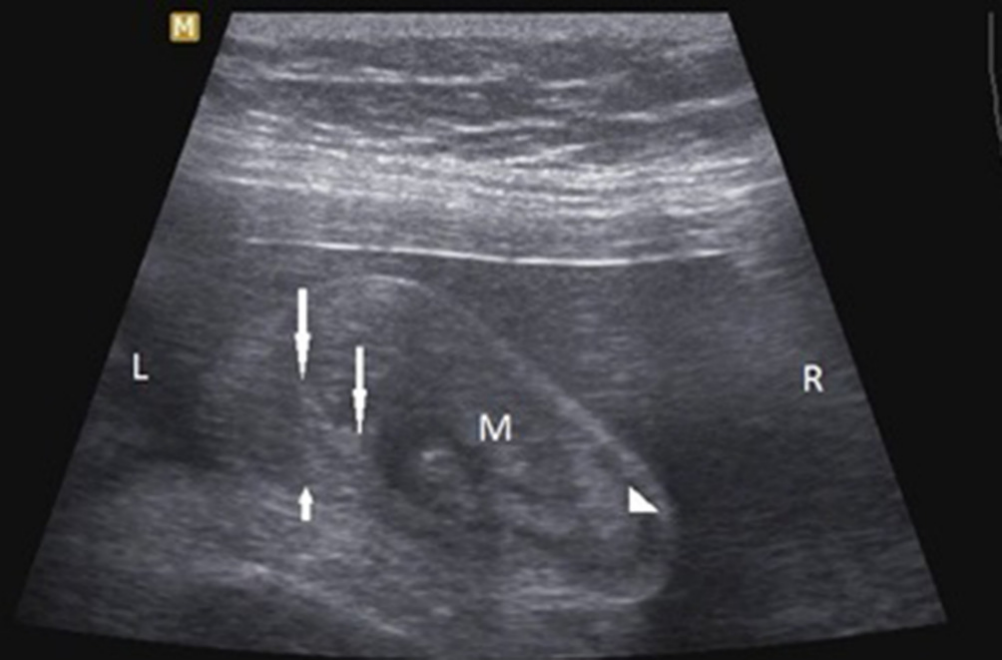
Longitudinal sonography of foetal scrotum showing a right-sided heterogeneous scrotal mass (**M**) and the displaced right testis (arrowhead). The median raphe (long arrows) is displaced to the left. The left testis (short arrow) is normal.

**Figure 2.**
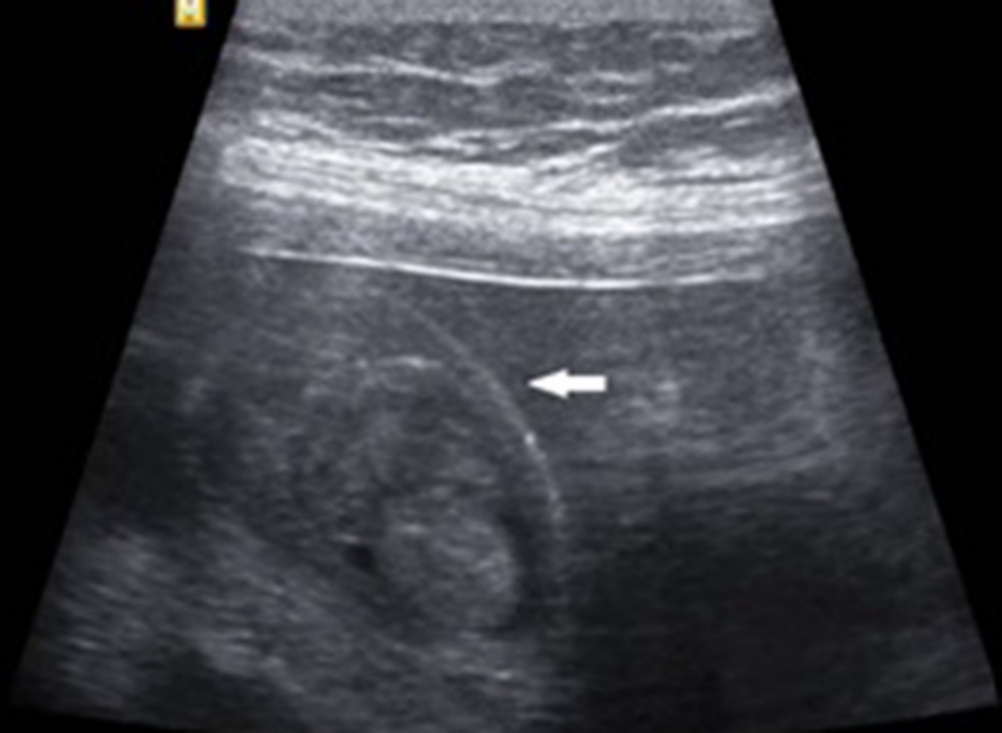
Longitudinal sonography of foetal scrotum showing an altered contour of the scrotum and movement of intrascrotal echoes. The surface of the scrotum is less convex (arrow) and concave (arrow in Figure 3) compared with Figure 1, where the surface is almost flat.

**Figure 3.**
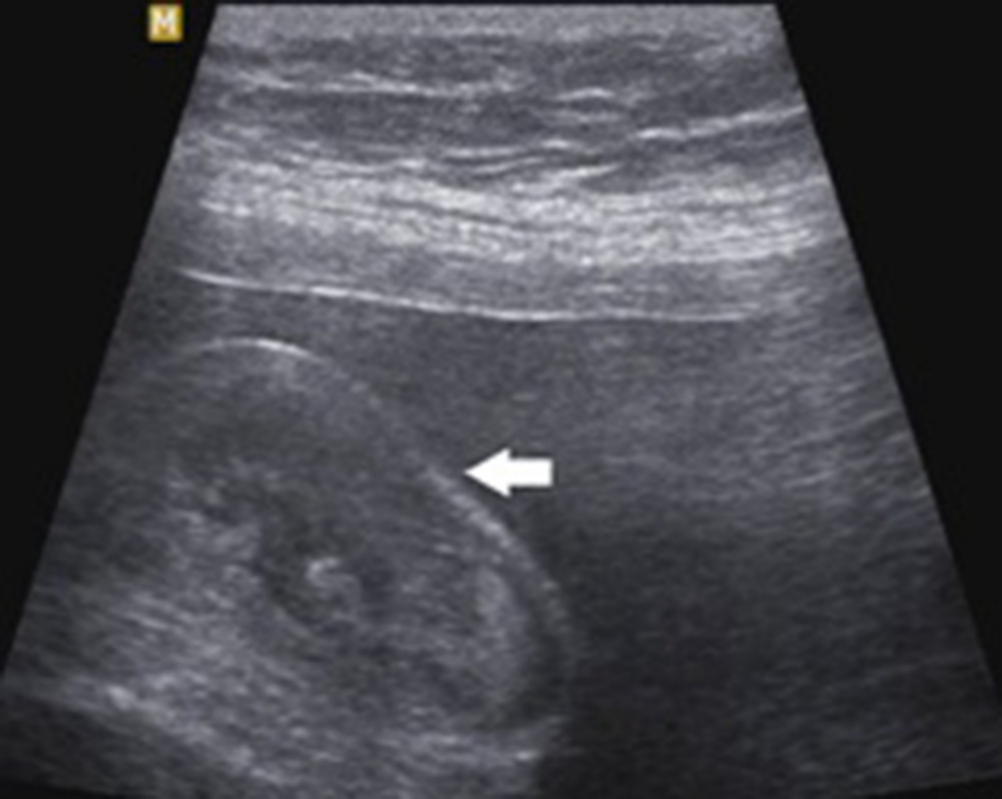
Longitudinal sonography of foetal scrotum showing concave surface (arrow).

Ultrasound examination showed a normal foetal biometry with adequate amniotic fluid. A mass was incidentally noted on the right side of the foetal scrotum measuring approximately 2.9 × 1.7 cm. The mass appeared heterogeneous in echotexture and showed echo movements within the lesion suggestive of peristalsis. The right testis was separately identified and displaced by the mass. The median raphe of the scrotum was displaced to the left and the left testis appeared normal (Figure 1). The contour of the scrotum was altered along with the movements of intrascrotal echoes (Figures 2 and 3). Based on these findings, a diagnosis of right inguinoscrotal hernia was made.

## Treatment

**Figure 4.**
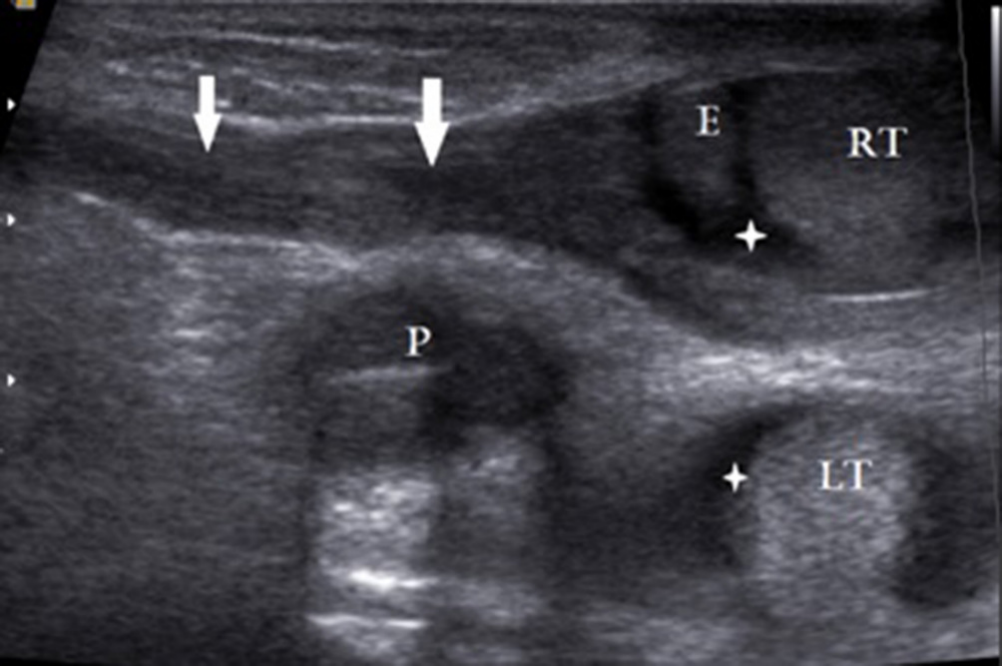
Oblique longitudinal sonogram of the right inguinal region of the male 1 week after birth showing a right patent processus vaginalis from the deep inguinal ring to the scrotum (arrows), epididymis (E) and right testis (RT). Echoes of a section of the root of the penis (P) and left testis (LT) are also seen on this plane. The asterisks indicate small hydroceles on both sides.

2 weeks later, she gave birth to a male baby by normal vaginal delivery. The birth weight of the baby was 3050 g. Surprisingly, clinical examination of the newborn revealed no evidence of scrotal swelling or hernia. Subsequent ultrasonography of the scrotum and inguinal region showed only patent processus vaginalis with a small communicating hydrocele on both sides (Figure 4). There was no hernia on ultrasonography and the testes were seen within the scrotum. Even during vigorous cry of the baby, there was no herniation seen through the deep inguinal ring. The parents were advised to bring the child for periodic follow-ups.

## Discussion

Inguinoscrotal hernia is usually diagnosed in the neonatal period or early infancy but is very rare in the foetus, as the foetal intra-abdominal pressure is equal to the amniotic fluid. Occurrence of an inguinoscrotal hernia after birth is aided by conditions that increase the intra-abdominal pressure such as vigorous cry, prematurity, chronic lung disease, ascites and bowel pathology.^1^ Herniation of the small bowel loops into the foetal scrotum occurs through the processus vaginalis. The processus vaginalis is an outpouch of the parietal peritoneum that evaginates into the scrotal fold during the second and third months of gestation. Between the seventh and ninth month of gestation, the testes descend into the scrotum through the processus vaginalis. Herniation of the bowel loops into the scrotum is expected to occur along with testicular descent.^2^ The processus vaginalis is obliterated nearing term, but remains patent at birth in 20% of the population and often closes during the first year of life. A patent processus vaginalis may result in failure of the testes to descend into the scrotum, communicating hydrocele or indirect inguinoscrotal hernia, but most of the cases of patent processes vaginalis remain asymptomatic throughout life.^2^

In case of a foetal scrotal mass, any echo movements within the mass should be looked for first. This feature is consistent with bowel peristalsis and is pathognomonic for herniation. Here we would like to recommend observing for alteration in the contour of the scrotum along with the intrascrotal echo movements. If this feature is present, it strongly augments the diagnosis. Sometimes bowel peristalsis is not seen at the time of examination and may be absent in case of obstruction, strangulation or ischemia.^1^ MRI may be useful to differentiate a herniated bowel from other mass if there is no peristalsis.^3^ Usually the testis on the involved side is displaced peripherally by the mass and there is no flow within the mass on colour Doppler studies. Sometimes blood flow signals from the herniated mesentery may be seen within the mass and this should not be confused with tumour vascularity. The gestational age at the time of diagnosis is usually not less than 26 weeks.^1^

Other causes of a foetal scrotal mass are hydrocele, testicular torsion, haemangioma and other solid tumours. Hydrocele is the most common scrotal pathology in the prenatal period.^4^ It can be easily identified, as it shows the typical appearance of a fluid-filled anechoic space within the scrotum, outlining the testicles. Meconium hydrocele appears as an echogenic scrotal mass and is usually seen with findings of meconium peritonitis such as ascites, abdominal mass, calcifications and meconium pseudocyst.^1^ Testicular torsion has been described prenatally with features of an enlarged testicle and epididymis with accumulation of two layers of haemorrhagic fluid, one between the visceral and parietal layers of the tunica vaginalis and another outside the tunica vaginalis (“double ring haemorrhage”).^5^ Haemangioma and teratoma are unusual foetal testicular mass lesions and they can be differentiated by their vascularity on colour Doppler examination.^1^

After the diagnosis of foetal inguinoscrotal hernia is made, the foetus should be monitored for signs of obstruction such as dilated bowel loops and polyhydramnios. If features of obstruction are present, the delivery should be planned according to the gestational age and other obstetric parameters by a team that includes a paediatric surgeon.^1^ In cases of uncomplicated foetal inguinoscrotal hernia, usually surgical correction will be carried out in the neonatal period, as there is a 30–40% risk of incarceration and possible strangulation in the first year of life.^6^

Very few cases of foetal inguinoscrotal hernia have been reported previously, of which all babies have undergone surgery except those who were dead.^7^ In our case, the foetal inguinoscrotal hernia had spontaneously regressed in the perinatal period and subsequent postnatal ultrasound revealed only a patent processus vaginalis and communicating hydrocele on both sides. Oudesluys-Murphy et al^8^ reported spontaneous regression of a clinically diagnosed inguinal hernia in pre-term female infants within the period of 2–6 months after birth. Spontaneous regression of foetal inguinoscrotal hernia in the perinatal period has not been reported so far.

## Learning points

Foetal inguinoscrotal hernia is unusual. Echo movements within the scrotal mass is the diagnostic sign of a foetal inguinoscrotal hernia and the diagnosis is augmented if there is contour alteration of the scrotal wall. Prompt diagnosis of foetal inguinoscrotal hernia and effective monitoring of signs of obstruction help to plan obstetric management. Spontaneous regression of uncomplicated foetal inguinoscrotal hernia during the perinatal period is also a possible outcome, as in our case. However, more studies are needed to validate this possibility.
